# Expression and function of Neuregulin 1 and its signaling system ERBB2/3 in the enteric nervous system

**DOI:** 10.3389/fncel.2015.00360

**Published:** 2015-09-23

**Authors:** Martina Barrenschee, Christina Lange, François Cossais, Jan-Hendrik Egberts, Thomas Becker, Thilo Wedel, Martina Böttner

**Affiliations:** ^1^Neurogastroenterology, Institute of Anatomy, Christian-Albrechts-University of KielKiel, Germany; ^2^Department of General, Thoracic, Transplantation and Pediatric Surgery, University Hospital Schleswig-Holstein, Campus KielKiel, Germany

**Keywords:** NRG1, HRG 1 beta, enteric nervous system, ERBB2, ERBB3

## Abstract

Neuregulin 1 (NRG1) is suggested to promote the survival and maintenance of the enteric nervous system (ENS). As deficiency in its corresponding receptor signaling complex ERBB2/ERBB3 leads to postnatal colonic hypo/aganglionosis we assessed the distributional and expressional pattern of the NRG1-ERBB2/ERBB3 system in the human colon and explored the neurotrophic capacity of NRG1 on cultured enteric neurons. Site-specific mRNA expression of the NRG1-ERBB2/3 system was determined in microdissected samples harvested from enteric musculature and ganglia. Localization of NRG1, ERBB2 and ERBB3 was determined by dual-label-immunohistochemistry using pan-neuronal and pan-glial markers. Morphometric analysis was performed on NRG1-stimulated rat enteric nerve cultures to evaluate neurotrophic effects. mRNA expression of the NRG1-ERBB2/3 system was determined by qPCR. Co-localization of NRG1 with neuronal or synaptic markers was analyzed in enteric nerve cultures stimulated with glial cell line-derived neurotrophic factor (GDNF). The NRG1 system was expressed in both neurons and glial cells of enteric ganglia and in nerve fibers. NRG1 significantly enhanced growth parameters in enteric nerve cell cultures and ErB3 mRNA expression was down-regulated upon NRG1 stimulation. GDNF negatively regulates ErbB2 and ErbB3 mRNA expression. The NRG1-ERBB2/3 system is physiologically present in the human ENS and NRG1 acts as a neurotrophic factor for the ENS. The down-regulation of ErbB3/ErbB2 in GDNF stimulated nerve cell cultures points to an interaction of both neurotrophic factors. Thus, the data may provide a basis to assess disturbed signaling components of the NRG1 system in enteric neuropathies.

## Introduction

Neuregulin 1 (NRG1), the best characterized member within the family of neuregulins, is a pleiotrophic growth factor that has been implicated in various functions in the nervous system e.g., nerve cell differentiation, neurite outgrowth and synapse formation (Falls, 2003; Mei and Nave, 2014). A variety of different isoforms are derived from the neuregulin gene, which can be distinguished based on differences in their NH2-terminal regions. The different NRG1 isoforms can be categorized into three groups: (i) type I NRG1 includes neu differentiation factor (NDF), the heregulins (HRGs) and acetylcholine receptor inducing activity (ARIA); (ii) type II NRG1 contains the glial growth factors (GGFs); and (iii) type III NRG1 comprises the sensory and motor neuron-derived factor (SMDF; Falls, 2003). NRG1 isoforms exert their effects through a heterodimeric complex consisting of members of the epidermal growth factor (EGF) receptor tyrosine kinases ErbB3/ErbB4 in the heart and ErbB2/ErbB3 in neural crest cells (Britsch et al., 1998; Garratt et al., 2000). All bioactive NRG isoforms contain an EGF-like core domain (α or β) that is sufficient for activation of ErbB receptor-tyrosine kinases (Buonanno and Fischbach, 2001).

Knock-out (KO) mice with targeted mutations of the Nrg1 gene show abnormalities in neural development of the central nervous system (CNS) and peripheral nervous system (PNS) in dependence on the deleted isoform, whereas NRG1 β isoforms (NRG1βs) are crucial for normal neuromuscular system development (Li et al., 2002; Falls, 2003).

The enteric nervous system (ENS) is one of the main divisions of the nervous system and comprises a complex network of ganglia and interconnecting nerve fibers that innervate the whole gastrointestinal (GI) tract and thus orchestrates the functions of the GI system (Furness, 2006). This neuronal network is arranged in two major plexus, i.e., the submucosal and myenteric plexus (MP; Wedel et al., 1999). Besides regulation of mucosal blood flow or ion and water transport, one major function exerted by the ENS is the regulation of GI motility, where the ENS acts as initiator that transmits the signals to the enteric smooth musculature via neurotransmitters and their receptors (Furness, 2012).

Consequently, ENS function can be impaired in enteric neuropathies underlying intestinal motility disorders as described for Hischsprung’s disease (HSCR; congenital colonic aganglionosis) slow transit constipation or diverticular disease (Knowles et al., 2009, 2010).

The importance of Nrg1 and its receptor signaling system ErbB2/ErbB3 in the ENS became evident when Nrg1, ErbB3 or ErbB2 KO mice were investigated. ErbB3^−/−^ mice exhibit severe neuropathies with complete loss of glia (Riethmacher et al., 1997) and reduced ganglia number in the duodenal gut (Erickson et al., 1997). Conditional ErbB2/Nestin-Cre mutant mice possess a dramatic reduction of enteric neurons and glia in the colon and display a phenotype that mimics human HSCR disease (Crone et al., 2003). In addition, a set of studies suggested an involvement of NRG1 signaling in the development and maintenance of the ENS. Nrg1 is expressed in mice and monkey intestinal mucosa (Meyer et al., 1997; Zhao, 2013) and mice enteric ganglia (Orr-Urtreger et al., 1993). The ErbB2/ErbB3 receptors are expressed in mouse neural crest cells that colonize the gut during development and in adult intestinal epithelia of both humans and mice (Paratore et al., 2002; Britsch, 2007).

However, a detailed characterization of the NRG1 system in the human colonic neuro-musculature system responsible for GI motility is still lacking. We therefore analyzed the site-specific gene expression levels, localization and distribution of type I NRG1 β1 (henceforth referred to as NRG1) and its receptors ERBB2 and ERBB3 in the musculature and myenteric plexus of the human colon. Furthermore we assessed the effect of NRG1 on differentiation parameters of enteric nerve cell cultures and analyzed the expression of Nrg1, ErbB2 and ErbB3 in enteric nerve cells stimulated with NRG1 or glial cell line-derived neurotrophic factor (GDNF), a typical neurotrophic factor for the ENS. Finally, we analyzed the localization and distribution of Nrg1 in enteric nerve cell cultures.

## Materials and Methods

### Human Tissue Source and Processing

Distal colonic segments were obtained from patients (*n* = 8, mean age: 75 years, three female, five males) who underwent partial colectomy for non-obstructing colorectal carcinoma. Colonic motility disorders and anorectal evacuation were excluded previously. Full-thickness specimens were harvested at a safe distance (>5 cm) from the tumor and immediately transferred for tissue processing to the laboratory. According to the guidelines (Knowles et al., 2009) this procedure refers to the common control tissue generation in gastroenterological neuromuscular pathology. The study of human tissue received approval from the Local Ethics Committee of the Faculty of Medicine, Christian Albrechts University of Kiel, Germany (B299/07).

After this surgical removal all of the specimens were transferred into PBS (phosphate-buffered saline, pH 7.2) to allow further dissection. Full-thickness tissue blocks (30 mm × 10 mm) with rectangle form were pinned out flat on a cork plate by fine needles without artificial stretching nor shortening, where the longer border of the tissue block was oriented vertical to the gut axis and thus corresponded to the cutting surface for histologic sections. Thus, myocytes of the circular muscle (CM) layer were cut along their longitudinal axis. For immunohistochemistry the tissue blocks were transferred into paraffin wax and cut in sections (6 μm) after fixation with 4% paraformaldehyde (in PBS) for 24 h and dehydration. For RNA analysis, tissue blocks were frozen in isopentane and stored at −70°C.

### Postnatal Myenteric Nerve Cell Cultures

Preparation of myenteric nerve cells was performed according to the method described previously (Schäfer et al., 1997). Briefly, after removing the small intestine from newborn Wistar rats (postnatal day 2), the tunica muscularis was stripped from the mucosa, incubated for 2 h at 37°C in Hanks’ Balanced Salt Solution (HBSS, Gibco Life Technologies/Invitrogen, Karlsruhe, Germany) that is free of Ca^2+^- and Mg^2+^ and completed with antibiotics and 1 mg/ml collagenase (SIGMA, Munich, Germany). Afterwards, fragments of MP were collected stereomicroscopically and incubated for at 37°C in trypsin/EDTA (0.125 mg/ml Gibco, Life Technologies, Germany) for 15 min to dissociate the plexus. For stopping this procedure trypsin/EDTA was replaced with fetal calf serum (FCS, Gibco, Life Technologies/Invitrogen, Karlsruhe, Germany). The cells were triturated, counted and seeded on 12-well-plates coated with poly-D-Lysin-(SIGMA)/Laminin (SIGMA, Munich, Germany) at a density of 100.000 cells/ml. Cells were incubated in defined medium consisting of Neurobasal A (Gibco, Life Technologies/Invitrogen, Karlsruhe, Germany) and B27 supplement (Gibco, Life Technologies/Invitrogen, Karlsruhe, Germany). Additionally, recombinant human NRG1-β1 (Thr176-Lys246, EGF Domain; R&D Systems, MN, USA) was added to a final concentration of 0 (control), 2 or 10 ng/ml or additionally for mRNA expression studies recombinant rat GDNF (Peprotech, Hamburg, Germany). Cells were cultured for 1 week where medium was changed every second day.

### Laser Capture Microdissection (LCM) and Dissection of Colonic Myenteric Plexus and Smooth Muscle Tissue

As described previously (Böttner et al., 2010), cryosections from isopentane frozen tissue blocks (14 μm) were placed on slides coated with polyethylene naphtalate (1 μm membrane, Carl Zeiss MicroImaging GmbH, Göttingen, Germany) and regions of interest were visualized by ultra-rapid (30 s) staining with cresyl violet according to P.A.L.M. RNA Handling Protocols (Zeiss MicroImaging, Göttingen, Germany). By inverse light microscopy (Axiovert, Zeiss, Jena, Germany), the myenteric ganglia were identified, laser-microdissected and collected by laser pressure catapulting (P.A.L.M. Microlaser Technologies, Bernried, Germany) in the lid of 0.5 ml reaction tubes. From each sample 2 mm^2^ ganglionic tissue was collected, dissolved in 200 μl RNA lysis buffer (PEQLAB, Erlangen, Germany) and stored at −70°C. Longitudinal and circular muscle tissue was collected under stereomicroscopic control from cryosections (20 μm) with excluding the myenteric ganglia by carefully dissecting the tissue with a scalpel. For each sample, the tissue of six cryosections were collected, dissolved in 200 μl RNA lysis buffer (PEQLAB, Erlangen, Germany) and storage was carried out at −70°C.

### Extraction of RNA and Reverse Transcription (RT)

Extraction of total RNA from human myenteric ganglia (MP), circular (CM) and longitudinal muscle (LM) was performed under usage of the peqGOLD MicroSpin Total RNA Kit (PEQLAB, Erlangen, Germany). Myenteric nerve cell cultures RNA was isolated by the Nucleospin XS kit (Macherey and Nagel, Düren, Germany) according to the manufacturer’s instructions. RNA was eluted in a volume of 15 μl H_2_O. Prior to reverse transcription (RT), contaminating genomic DNA was digested for 15 min at room temperature using 1.5 U of DNase I (Sigma-Aldrich, Munich, Germany). RT was carried out in a total volume of 30 μl containing 375 ng random hexamer primer (GE Healthcare, Freiburg, Germany), 0.5 mM dNTPs (Promega, Mannheim, Germany), 0.01 M DTT, 1 × reaction buffer, and 150 U Superscript II Reverse Transcriptase (Invitrogen, Karlsruhe, Germany). Annealing, elongation, and denaturation were carried out at 25°C for 10 min, 42°C for 50 min and at 70°C for 15 min, respectively.

### Quantitative PCR (qPCR)

Quantitative PCR (qPCR) reactions were performed in 96 well plates in duplicates. Each reaction (20 μl) contained 2 μl of total cDNA, 900 nM primers, 225 nM hybridization probe and 1 × qPCR Master Mix Plus (Eurogentec, Cologne, Germany). qPCR product accumulation was monitored by the ABI Prism 7700 cycler (TaqMan, Applied Biosystems, CA, USA) for 45 cycles. Cycle contained a denaturation phase of 15 s at 95°C and an elongation phase of 1 min at 60°C. The data were normalized to the expression levels of the housekeeping gene hypoxanthine-guanine phosphoribosyltransferase (HPRT), expressed as relative mRNA expression and presented as mean ± SEM. Forward and reverse primers and probes are listed below.

#### Primers Amplifying Human Sequences

*NRG1 type I HRG β1* (NM_013956.3): forward primer: 5′-*atggaggcggaggagctgta*-3′, reverse primer: 5′-*ttgcagtaggccaccacaca*-3′, probe: 5′-*tgaccataaccggcatctgcatcgc*-3′; *ERBB2* (NM_004448.2): forward primer: 5′-*ggaagtacacgatgcggagact*-3′, reverse primer: 5′-*tctctttcaggatccgcatctg*-3′, probe: 5′-*tggagccgctgacacctagcgga*-3′; *ERBB3* (NM_001982.3): forward primer: 5′-*tgccatcttcgtcatgttgaac*-3′, reverse primer: 5′-*tcaatataaacaccccctgacagaa*-3′, probe: 5′-*agctccgcttgactcagctcaccga*; HPRT (NM_000194.2, house-keeping gene): forward primer: 5′-*tgaacgtcttgctcgagatgtg*-3′, reverse primer: 5′-*ccagcaggtcagcaaagaattt*-3′, probe: 5′-*tgggaggccatcacattgtagcc*-3′.

#### Primers Amplifying Rat Sequences

*Nrg1 type I Hrg-β1* (AY973244.1) forward primer: 5′-*ctaccagaagagggtgctgacaa*-3′, reverse primer: 5′-*gccgctgcttcttggtttt*-3′, probe: 5′-*ctgctggtggtcggcatcttgtgtg*-3′; *Erbb2* (NM_017003.2): forward primer: 5′-*gctgctgcaggaaactgagttag*-3′, reverse primer: 5′-*ccttccttagctccgtctcttttag*-3′, probe: 5′-*ctgacgcccagcggagcaatgc*-3′; *Erbb3* (XM_006240755.1) forward primer: 5′-*cgaggagatgcgagctttcc*-3′, reverse primer: 5′-*aaagcctgctgtgccagtaatc*-3′, probe: 5′-*ccccatgttcgttatgcccgcct*; *hprt* (NM_012583.2, house-keeping gene): forward primer: 5′-*cgccagcttcctcctcaga-*3′, reverse primer: 5′-*ggtcataacctggttcatcact*-3′, probe: 5′-*ttttcccgcgagccgaccgg*-3′.

### Immunohistochemistry

#### Conventional Immunohistochemistry of NRG1, ERBB2 and ERBB3 in Human Colonic Tissue

Immunoreactive signals were uncovered under usage of the avidin-biotin-complex system (Vectastain Elite ABC Kit, Vector Laboratories, Burlingame, CA, USA). To block the activity of endogenous peroxidase paraffin embedded tissue sections were incubated with 3% hydrogen peroxide briefly, rinsed in TBS-buffer (TRIS-buffered saline; 10 mM TRIS, 50 mM NaCl, pH 7.4) and pretreated with citrate buffer (pH 6.0) in a 95°C water bath for 20 min. Thereafter, samples were incubated overnight with a polyclonal rabbit-anti-NRG1 β1 antibody (HRG β1, 1:500, antibodies-online.com, Aachen, Germany; immunogen sequence: KKPGKSELRINKAS), a polyclonal rabbit-anti-ERBB2 antibody (1:1000, antibodies-online.com, Aachen, Germany) or a polyclonal rabbit-anti-ERBB3 antibody (1:2000 antibodies-online.com, Aachen, Germany) respectively diluted in antibody diluent (Invitrogen, Karlsruhe, Germany) and incubated with biotinylated goat anti-rabbit IgG (1:400, DAKO, Hamburg, Germany) for 45 min. After washing three times with TBS, sections were incubated for 45 min with an avidin-biotin-complex (Vectastain ABC Standard, Vector Laboratories, Burlingame, CA, USA) conjugated with horseradish peroxidase. 3,3′-diaminobenzidine (DAKO, Hamburg, Germany) was used as chromogen. Sections were counterstained with Meyer’s hematoxylin and negative controls were creating by omission of the primary or secondary antibody. Analysis was carried out with a light optical microscope (Nikon 6000, Nikon, Tokyo) coupled to a digital camera (Digital Sight, Nikon, Tokyo). Data acquisition was performed with NIS-Elements BR 3.2 software (Nikon, Tokyo).

#### Dual-Label Immunohistochemistry of NRG1, ERBB2 and ERBB3 with the Pan-Neuronal Marker PGP 9.5 or the Glial Cell Marker S100b

Pretreatment of paraffin embedded tissue sections was carried out with citrate buffer (pH 6.0, 95°C water bath) for 25 min followed by overnight incubation with either rabbit-anti-HRG β1 (NRG1 1:200, antibodies-online.com, Aachen, Germany), rabbit-anti-ERBB2 (1:500, antibodies-online.com, Aachen, Germany) or rabbit-anti-ERBB3 antibody (1:1000 antibodies-online.com, Aachen, Germany) respectively, diluted in antibody diluent (Invitrogen, Karlsruhe, Germany) as primary antibodies. After washing with TBS, sections were incubated with goat anti-rabbit alexaFluor488 antibody, diluted in antibody diluent (1:250, Invitrogen, Karlsruhe, Germany) as secondary antibodies for 2 h at room temperature. Enteric neuron visualization was realized by co-incubating the sections with mouse-anti-PGP 9.5 (1:1000, Acris, Herford, Germany), enteric glia was visualized by co-incubating the sections with mouse-anti-S100b (1:1000, Merck Millipore, Billerica, MA, USA). Secondary antibody incubation was carried out with goat-anti-mouse AlexaFluor546 antibody (1:250; Invitrogen, Karlsruhe, Germany) for 2 h at room temperature. DAPI staining of sections to visualize cellular nuclei (Roche, Mannheim, Germany) was carried out finally. Fluorescence signals were detected with a fluorescence microscope (Axiovert 200M), (Zeiss, Göttingen, Germany), linked to an Axiocam digital camera. Data were analyzed with the Axiovision software (Zeiss, Göttingen, Germany). Merging of immunoreactive signals was processed by using the Zeiss co-localization tool (Zeiss, Göttingen, Germany).

#### Immunocytochemistry of NRG1 in Enteric Nerve Cell Cultures

Dual-label immunocytochemistry for NRG1 was performed with PGP 9.5 or SNAP-25 in cells that were stimulated for growth with 50 ng/ml GDNF (1 week). Cells fixing was realized by incubating the specimens with 4% paraformaldehyde for 30 min. Cells were permeabilized for 10 min with methanol and blocked with normal goat serum (1:10, DakoCytomation, Glostrup, Denmark) for 30 min. Incubation with rabbit-anti-HRG β1 (NRG1 1:200, antibodies-online.com, Aachen, Germany) and either mouse anti-SNAP-25 antibody (1:10000, LIFESPAN, Seattle, WA, USA) or mouse anti-PGP 9.5 antibody (1:1000, Acris, Herford, Germany) for 1 h was performed, followed by incubation with the secondary antibodies, goat anti-rabbit-AlexaFluor488 (1:250, Invitrogen, Life technologies, Carlsbad, CA, USA) and goat anti-mouse-AlexaFluor546 (1:250, Invitrogen, Life technologies, Carlsbad, CA, USA). To visualize cell nuclei specimens were counterstained with DAPI (Roche, Mannheim, Germany). Analysis was carried out under usage of a fluorescent microscope (Axiovert 200 M, Zeiss, Göttingen, Germany) that was linked to an Axiocam digital camera (Axiocam, Zeiss, Göttingen, Germany). The acquisition of data was performed with the Axiovision software (Zeiss, Göttingen, Germany).

### Morphometric Analysis of Enteric Nerve Cell Cultures

#### Immunocytochemistry in Enteric Nerve Cell Cultures

Cells were fixed for 30 min with 4% paraformaldehyde, permeabilized for 10 min with methanol and treated for 10 min with 3% H_2_O_2_. After blocking of unspecific background with normal goat serum (1:10, DakoCytomation, Glostrup, Denmark) for 30 min, incubation with a mouse anti-PGP 9.5 antibody (1:1000, Acris, Herford, Germany) for 1 h was performed, followed by incubation with a biotinylated secondary antibody (goat anti-mouse IgG, 1:400) for 45 min and treatment with an avidin-biotin-complex (Vectastain ABC Standard, Vector Laboratories, Burlingame, CA, USA) conjugated with horseradish peroxidase for 45 min. Antibody binding was visualized with 3,3′-diaminobenzidine (DAKO, Hamburg, Germany).

#### Technical Devices, Software

Morphometric analysis was carried out by using a light optical microscope (Nikon 6000, Nikon, Tokyo) coupled to a digital camera (Digital Sight, Nikon, Tokyo, Japan). Three randomly chosen optical fields were recorded per cell culture approach (200x magnification). Data acquisition on captured images was performed with NIS-Elements BR 3.2 software (Nikon, Tokyo, Japan), transferred into Excel 2010 (Microsoft Corporation, Redmond, WA, USA) and further processed for statistical analysis and data plotting (Prism^TM^, GraphPad, San Diego, CA, USA).

#### Growth Parameters

*Nerve fibers* were retraced per image, the sum of nerve fiber length was calculated in three randomly assigned optical fields and the mean value was calculated. Data were presented as mean of total nerve fiber length per optical field.

*Branching points and ganglion-like aggregates* were counted in three randomly assigned optical fields and the mean value was calculated. Data were presented as mean branching points per optical field and mean number of ganglion-like aggregates per optical field. Ganglion-like aggregates are defined as a cluster of neurons creating assemblage points, resembling enteric ganglia (Figure 7). Experiments were carried out in nine replicates (2 and 20 ng/ml NRG1 vs. control).

### Statistical Analysis

Data were analyzed by Mann–Whitney U test (Prism^TM^, GraphPad, Sand Diego, CA, USA) followed by *post hoc* test with the FDR method (false-discovery rate by using R 2.13.1 (R-Core-Team, 2012). Differences were determined significant if *p* < 0.05.

## Results

### Site-Specific Gene Expression of ERBB2 and ERBB3 and their Corresponding Ligand NRG1 in the Human Tunica Muscularis

**Figure 1:**
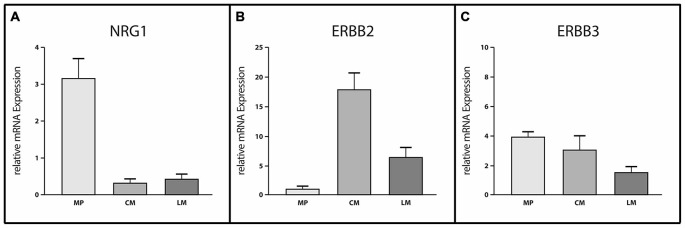
**Site-specific mRNA expression of Neuregulin 1 (NRG1), ERBB2 and ERBB3 in myenteric ganglia and smooth muscle layers of the adult human colon.** Analysis of site-specific mRNA expression profiles in microdissected myenteric plexus (MP), circular muscle (CM) and longitudinal muscle (LM) revealed myenteric ganglia as the main source of NRG1 **(A)** whereas ERBB2 **(B)** is mostly expressed in the muscle layers. ERBB3 mRNA levels **(C)** are reduced in LM compared to MP or CM where expression levels were approximately equal. Levels of mRNA were detected by reverse transcription (RT)-qPCR and expression of genes of interest was normalized to the mRNA expression of the house-keeping gene HPRT. Data are shown as mean ± SEM, *n* = 6–8 per experimental group.

In order to analyze the expression pattern of the NRG1-ERBB2/3 system in the human colon, we performed quantitative real time PCR experiments of RNA isolated from CM, LM and MP, separated per laser-capture microdissection (Figure 1). mRNA expression of ERBB2, ERBB3 and their corresponding ligand NRG1 was detected in all three tissues but differs in their expression levels. Analysis of expression profiles revealed up to sixfold higher expression levels of NRG1 (Figure 1A) in MP compared to both muscle layers, that exhibited nearly the same expression levels whereas ERBB2 (Figure 1B) exhibited more pronounced expression in both muscle layers compared to MP. mRNA expression of ERBB2 was up to sixfold higher in LM and up to 17-fold higher in CM when compared to MP. In contrast ERBB3 mRNA showed approximately equal expression levels in MP and CM but twofold reduced expression level in LM (Figure 1C).

### Conventional Immunocytochemistry of NRG1, ERBB2, and ERBB3 in the Human Tunica Muscularis

**Figure 2:**
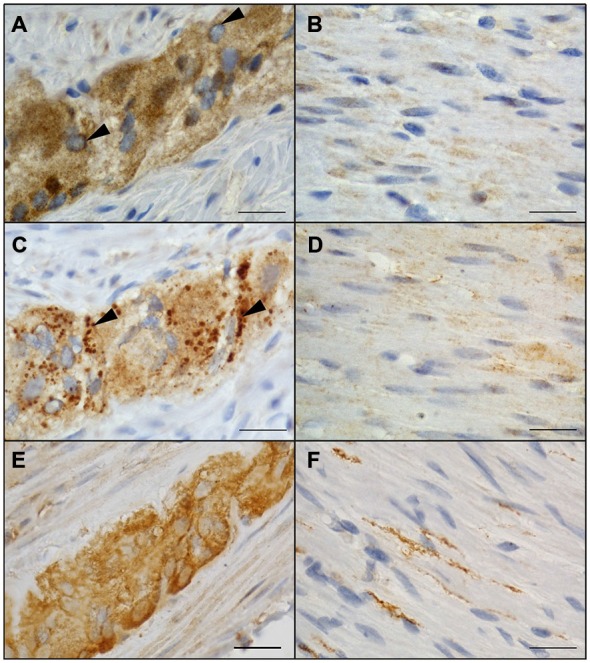
**Localization of NRG1, ERBB2 and ERBB3 in the colonic myenteric plexus and circular musculature of the adult human colon.** NRG1 immunopositive signals were identified in myenteric ganglia with robust staining in neuronal somata and weaker signals in the surrounding neuropil **(A)**. Perinuclear staining (arrowhead) was detected on all glial nuclei **(A)**. In the circular musculature, smooth NRG1 immunoreactivity was observed as faint signals with homogenous distribution **(B)**. ERBB2 immunoreactivity in myenteric ganglia **(C)** appeared to be less pronounced compared to the surrounding neuropil but showed additional strong granular staining (arrowheads) confined to cell somata. In the circular musculature, ERBB2 immunoreactivity **(D)** displayed slight signals similar to NRG1 and also with uniform distribution. ERBB3 immunoreactivity was also found in myenteric ganglia **(E)** accumulated in neuronal somata of singe neurons and also observed area-wide throughout the ganglia as well as in nerve fibers running within the CM layer **(F)**. Blue color: haematoxylin counterstain. Bars: 20 μm.

Localization of NRG1, ERBB2, and ERBB3 in the human tunica muscularis was determined by conventional immunohistochemistry on colonic full-thickness sections, using 3,3′-diaminobenzidine as cromogen. Consistent with the findings of gene expression studies, NRG1 immunoreactivity was detected in ganglia of the myenteric plexus (Figure 2A) and circular musculature (Figure 2B), where signals was distributed in neuronal somata large-area wide with a higher density in the neuronal nucleus. NRG1 immunoreactivity exhibited also strong signals in some but not all glial nuclei but perinuclear signals were detected on most of the glial nuclei (Figure 2A, arrowhead). The weakest signals in the myenteric plexus were found in the neuropil. The circular musculature exhibited faint NRG1 immunopositive signals (Figure 2B) throughout the cell bodies, but also signals were detected in some nuclei. ERBB2 immunoreactivity was also detected in both the myenteric plexus (Figure 2C) and the circular musculature (Figure 2D). Punctuate staining was observed throughout the myenteric ganglia with higher density in neuronal somata compared to the neuropil (Figure 2C, arrowhead). In the circular musculature, the ERBB2 immunoreactive pattern was similar to that demonstrated for NRG1 (Figure 2D), but in difference it exhibited stronger area-wide signals, compared to the NRG1 immunoreactive pattern. ERBB3 immunoreactivity was also found in the MP (Figure 2E). Immunoreactive signals were detected in neuronal somata and to a smaller extend throughout the ganglionic neuropil, however in neuronal nuclei, ERBB3 immunoreactive was excluded. ERBB3 immunoreactivity displayed also signals in the nerve fibers within the circular musculature, but it was also found in a few cell nuclei (Figure 2F).

### Dual-Label Immunocytochemistry of NRG1, ERBB2 and ERBB3 with the Pan-Neuronal Marker PGP 9.5 and the Pan-Glial Marker S100b in the Human Tunica Muscularis

**Figure 3:**
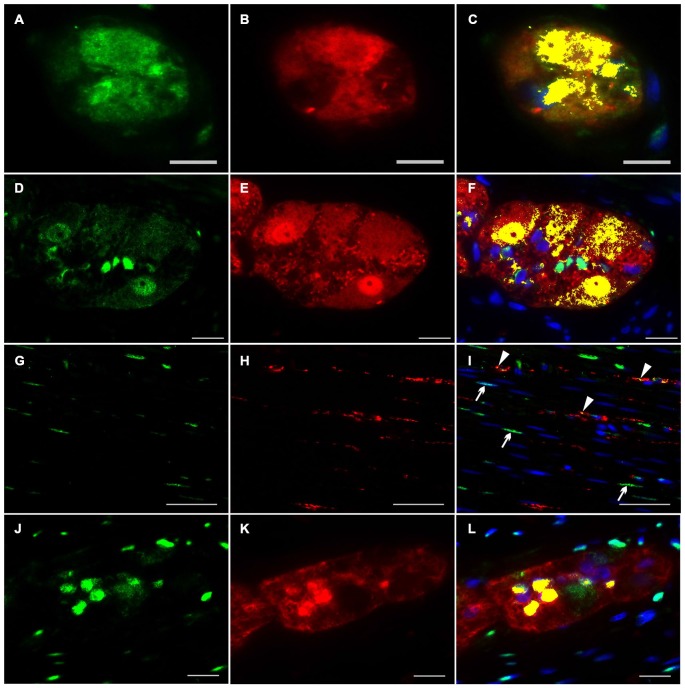
**Co-localization of NRG1 and PGP 9.5 or S100b in the human colon.** NRG1 immunoreactivity (green) was detectable in submucosal **(A,C)** and myenteric ganglia **(D,F)** visualized by the pan-neuronal marker PGP 9.5 (red, **B,E**), in the circular musculature **(G–I)** as well as in myenteric ganglia visualized with the pan-glial marker S100b (red, **K,L**). NRG1 immunoreactivity in both submucosal and myenteric ganglia displayed predominant accumulation in gilal and neuronal nuclei and neuronal somata but also exhibited faint signals in the neutropil. In the circular musculature, NRG1 signals were found in nerve fibers (**I**, arrowhead) and also in some muscle cell nuclei. (**I**, arrow) Co-localization (yellow) of NRG1 and PGP 9.5 is shown in merged panels **(C,F,I)**. Co-localization (yellow) of NRG1 and S100b is shown in merged panel **(L)**. Blue color: DAPI staining of nuclei. Bars **(A–C)** = 10 μm; **(D–F)**: 20 μm; **(G–I)**: 50 μm, **(J–L)**: 20 μm.

**Figure 4:**
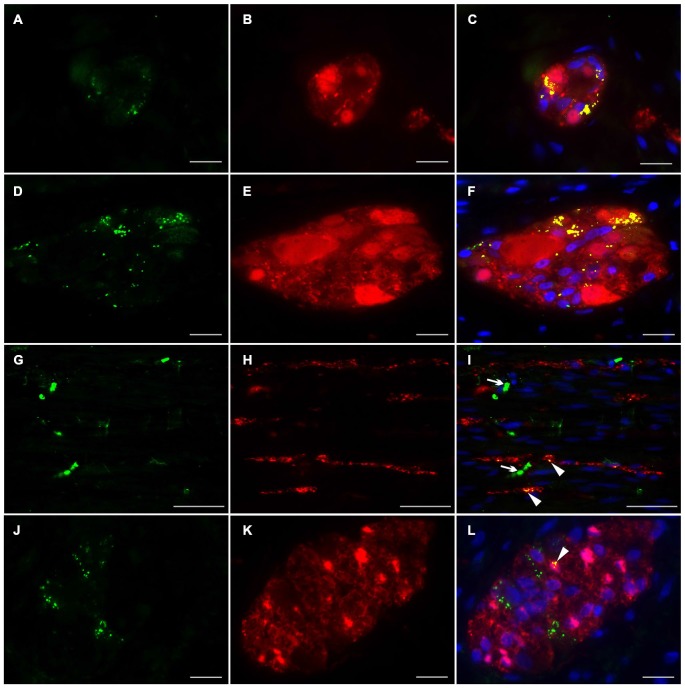
**Co-localization of ERBB2 and PGP 9.5 or S100b in the human colon.** ERBB2 immunoreactivity (green) was observed in both submucosal **(A,C)** myenteric ganglia **(D,F)** visualized by the pan-neuronal marker PGP 9.5 (red, **B,E**), in the circular musculature **(G–I)** as well as in myenteric ganglia visualized with the pan-glial marker S100b (red, **K,L**). ERBB2 showed patchy and granular immunoreactive signals distributed in the neuronal somata and in the neuropil of the submucosal and myenteric plexus and also exhibited faint signals in a few glial nuclei (**L**, arrowhead). In the circular musculature **(G–I)** ERBB2 immunoreactivity was observed as inhomogeneous point-shaped signals with minor extend also in enteric nerve fibers (**G,I**, arrowhead in **I**) and muscle cells (**G,I**, arrow in **I**). Co-localization (yellow) of ERBB2 and PGP 9.5 is shown in merged figures **(C,F,I)**. Co-localization (yellow) of NRG1 and S100b is shown in merged panel **(L)**. Blue color: DAPI staining of nuclei. Bars **(A–F)**: 20 μm; **(G–I)**: 50 μm, **(J–L)**: 20 μm.

**Figure 5:**
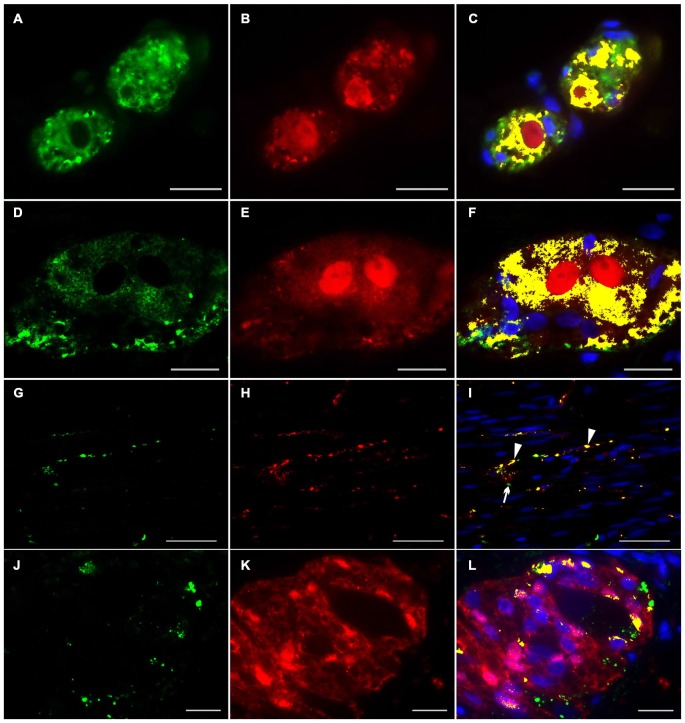
**Co-localization of ERBB3 and PGP 9.5 or S100b in the human colon.** ERBB3 (green) was localized in enteric nerve cells visualized by the pan-neuronal marker PGP 9.5 (red, **B,E,H**) of submucosal **(A,C)** and myenteric ganglia **(D,F)**, in nerve fibers running within the circular musculature **(G,I)** as well as in myenteric ganglia visualized with the pan-glial marker S100b (red, **J,K**). ERBB3 immunoreactive signals were accumulated in somata of single neurons and glial cells and also observed area-wide throughout the ganglia **(C,F,L)**. In the circular musculature **(G–I)** most ERBB3 immunoreactive signals were restricted to nerve fibers (**I**, arrowhead). Co-localization (yellow) of NRG1 and PGP 9.5 is shown in merged figures **(C,F,I)**. Co-localization (yellow) of NRG1 and S100b is shown in merged figures **(L)**. Blue color: DAPI staining of nuclei. Bars **(A–F)**: 20 μm; **(D–F, G–I)**: 50 μm, **(J–L)**: 20 μm.

To investigate the distributional expression pattern of NRG1, ERBB2 and ERBB3 in neuronal structures, we performed dual-label immunocytochemistry with PGP 9.5 as pan-neuronal marker. For investigation the distribution of the NRG1-System in glial cells co-localization experiments were performed with S100b as pan-glial marker. NRG1 immunoreactive signals were detected in the submucosal (Figures 3A–C) and myenteric plexus (Figures 3D–F) in neuronal somata and nuclei. As shown with conventional immunohistochemistry before, NRG1 immunoreactivity was also detected in the neuropil and as perinuclear signals around a few glial nuclei. In addition in the myenteric plexus NRG1 exhibited some strong rounded signals, that did not co-localized with PGP 9.5, but co-localized with the pan-glial marker S100b, indicating, that some, but not all glial cells express NRG1 (Figures 3J–L). NRG1 immunoreactivity was also detected in the circular musculature, with weaker signals in nerve fibers (Figures 3G–I, arrowhead in I). Strong immunoreactivity was observed in some muscle cell nuclei (Figures 3G–I, arrowhead in I). ERBB2 immunoreactivity was found as punctuate staining in the neuronal somata of some, but not all myenteric or submucosal neurons. The neuropil exhibited minor extend of punctuate ERBB2 immunoreactivity (Figures 4A–F) and ERBB2 showed slight signals in some glial nuclei (Figures 4J–L, arrowhead in L). Some, but not all nerve fibers in the circular musculature displayed ERBB2 immunoreactivity with punctuate pattern (Figures 4G–I, arrowhead in I). In addition, some muscle cells also exhibit ERBB2 immunoreactive signals (Figures 4G,I, arrow). Strong ERBB3 immunoreactive signals, that co-localized with the pan-neuronal marker PGP 9.5 were found area-wide in submucosal and myenteric neuronal somata and the neuropil (Figures 5A–F), whereas neuronal and glial cell nuclei where generally excluded. Within the circular musculature, ERBB3 co-localized with PGP 9.5, indicating ERBB3 presence also in nerve fibers (Figures 5G–I). In addition, ERBB3 immunoreactivity co-localized to some extend with S100b (Figures 5J–L) in the myenteric plexus, indicating that ERBB3 is also expressed in some glial cells.

#### Localization of Nrg1 in Enteric Nerve Cell Cultures

**Figure 6:**
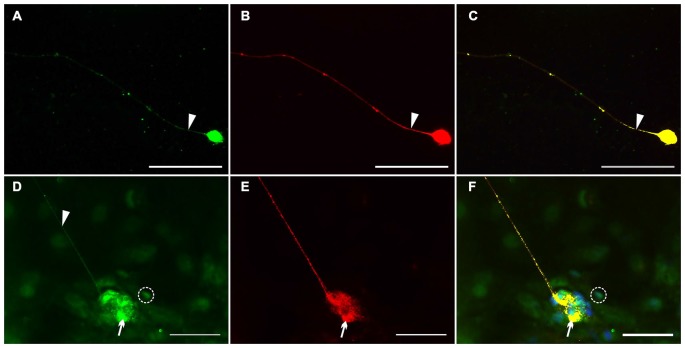
**Localization of NRG1 in single myenteric neurons and ganglion-like aggregates.** Dual-label immunocytochemistry for NRG1 **(A–C)** with PGP 9.5 **(B)** and NRG1 **(D–F)** with SNAP-25 **(E)** was performed after glial cell line-derived neurotrophic factor (GDNF) treatment (50 ng/ml) for 1 week. NRG1 co-localization with PGP 9.5 was found in neuronal somata and processes of single enteric neurons **(A,C)**. In nerve cell aggregates **(D–F)**, NRG1 co-localization with SNAP-25 **(F)** was found in the neuropil (arrows) and neuronal processes (arrowhead in **D**). NRG1 immunoreactive signals was also found in some glial cells (**D,F**, dashed circle). Blue color: DAPI staining of nuclei. Scale bars = 50 μm.

To assess the Nrg1 expression *in vitro*, we performed dual-label immunocytochemistry on myenteric nerve culture with PGP 9.5 as pan-neuronal marker for visualization of neuronal somata and processes in single neurons (Figures 6A–C) and with synaptosomal-associated protein 25 (SNAP-25), a synaptic vesicle marker expressed in enteric ganglion-like aggregates and neuronal processes (Figures 6D–F). PGP 9.5 displayed an uniform labeling of neuronal elements (somata and processes) of single myenteric neurons (Figures 6B,C), while NRG1 immunoreactivity was found as homogeneous signal in neuronal somata (Figures 6A,C) and slight granular staining in nerve processes (arrow) that all co-localize with PGP-9.5 (Figure 6C). In myenteric ganglion-like aggregates, NRG1 co-localizes with the vesicle marker SNAP-25 in the neuropil (Figure 6D, arrow) and neuronal processes (Figure 6D, arrowhead). NRG1 immunoreactive signals were also found in some glial cells (Figures 6D,F, dashed circle) and adjacent muscle cells.

#### Effects of NRG1 on Neuronal Network Formation of Cultured Enteric Neurons

**Figure 7:**
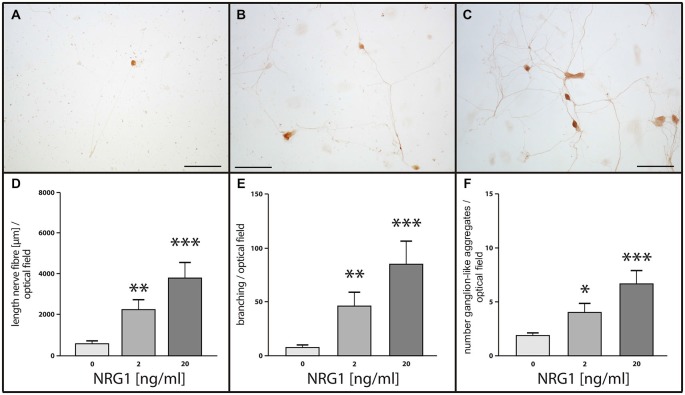
**Effects of NRG1 on nerve fiber length, number of ganglion-like aggregates and branching point quantity of cultured myenteric neurons.** Representative photographs of rat enteric nerve cells cultured for 1 week without NRG1 (**A**, control), with 2 ng/ml NRG1 **(B)** or with 20 ng/ml NRG1. **(C)** Control cultures showed only limited outgrowth, branching of neurites and number of ganglion-like aggregates, whereas treatment of cultures with NRG1 exhibited an enhancement of neurite outgrowth, increased number of neurite branches and ganglion-like aggregates. Morphometric analysis **(D–F)** confirmed the significant increase of total neurite length **(D)**, the number of neurite branches **(E)** and the number of ganglion-like aggregates **(F)** by NRG1 compared to controls. Bars: 100 μm. Visualization of neuronal structures was carried out by immunocytochemistry for the pan-neuronal marker PGP 9.5. Data are shown as mean ± SEM, *n* = 9 per experimental group, *p < 0.05, **p < 0.01, ***p < 0.001 vs. control.

To assess the impact of NRG1 on neuronal network formation and neurite outgrowth of enteric neurons, primary cultures were treated with NRG1 (2, 20 ng/ml) and immunocytochemistry for the pan-neuronal marker PGP 9.5 was performed. Nerve fiber length, number of branching points and number of ganglion-like aggregates resembling enteric ganglia were measured (Figures 7A–C). After 1 week in culture, only sparse nerve fibers could be identified in untreated cultures (control; Figure 1A), whereas NRG1-treated cultures displayed a dose-dependent increase of full nerve fiber length, with median nerve fiber length of 550 μm for the controls, 2200 μm for cultures stimulated with 2 ng/mL NRG1 and 3700 μm for cultures stimulated with 20 ng/mL NRG1 (Figure 7D). NRG1 stimulated myenteric cell culture developed neuronal networks dependent of NRG1 concentration as characterized by the number of branching points per optical field with a median value of 7 for the controls, 50 for cultures stimulated with 2 ng/mL NRG1 and 92 for cultures stimulated with 20 ng/mL NRG1 (Figure 7E). Ganglion-like aggregates are also increased in number in dependence of NRG1 concentration with median numbers of 2, 4 and 7 for controls, cultures stimulated with 2 ng/ml and cultures stimulated with 20 ng/ml NRG1, respectively (Figure 7F) and also exhibited in some cases a greater size (Figure 7C).

#### Effects of NRG1 and GDNF on Nrg1, ErbB2 and ErbB3 mRNA Expression in Cultured Enteric Neurons

**Figure 8:**
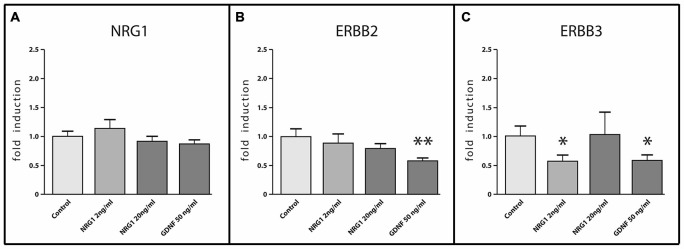
**mRNA expression of NRG1, ERBB2 and ERBB3 in enteric nerve cell cultures in response to NRG1 or GDNF treatment.** NRG1 mRNA expression in enteric nerve cells were not regulated by NRG1 or GDNF stimulation **(A)**, whereas NRG1 decreased mRNA levels of ERBB3 **(C)** and GDNF decreased mRNA levels of both ERBB2 and ERBB3 **(B,C)**. Enteric nerve cells were cultured for 6 days. Expression levels of the target gene were normalized to expression of the house-keeping gene HPRT. Data are shown as mean ± SEM, *n* = 15–18 per experimental group, *p < 0.05, **p < 0.01 vs. control.

To investigate the effect of NRG1 and the well-known enteric neurotrophic factor GDNF on gene regulation of the enteric NRG1 system itself, mRNA expression of *Nrg1*, *ErbB2* and *ErbB*3 were measured by RT-qPCR in rat enteric nerve cell cultures treated with increasing concentrations of NRG1 of 2 ng/ml and 20 ng/ml and 50 ng/ml GDNF for 1 week (Figure 8). Neither NRG1 nor GDNF treatment regulated the *Nrg1* mRNA enteric nerve cultures (Figure 8A) and also no regulation of *ErbB2* was observed upon NRG1 treatment (Figure 8B). However, 50 ng/ml GDNF administration significantly down-regulated *ErbB2* up to 50% as compared to untreated controls (Figure 8B), and the expression of *ErbB3* mRNA was down-regulated up to 50% upon both 2 ng/ml NRG1 and 50 ng/ml GDNF (Figure 8C), while 20 ng/ml NRG1 treatment had no significant effect (Figure 8C).

## Discussion

Although expression and localization of NRG1 and its corresponding receptors have been described in various tissues and organs, their distribution in main components of the colonic neuromuscular system has not been characterized yet. Thus, we investigated the site-specific gene expression and localization of NRG1 and ERBB2/ERBB3 in the human colon, and further analyzed the neurotrophic capability of NRG1 on cultured enteric neurons.

First, we showed differential expression and distribution of NRG1 and its signaling system ERBB2/ERBB3 in the human colonic tunica muscularis. Expression of NRG1 was predominant in myenteric ganglia and NRG1 co-localized with PGP 9.5 and the synaptic vesicle marker SNAP-25 in enteric nerve cell cultures. ERBB2 was highest expressed in the musculature. Treatment of enteric nerve cell cultures with NRG1 significantly enhanced neuronal growth. GDNF treatment led to down-regulation of ErbB2/ErbB3 mRNA expression in enteric nerve cell culture.

### Expression and Localization of NRG1, ERBB2 and ERBB3 in the Human Colon and Enteric Nerve Cells

NRG1 is expressed mainly in the myenteric plexus. This observation is in line with its abundant expression in the CNS and points to its dominant role in neurotransmission, neuroplasticity and glial-neuron interaction, as described before (Marchionni et al., 1999; Mei and Xiong, 2008; Ting et al., 2011). In the ENS, NRG1 type I mRNA transcripts were previously found in the normoganglionic segment of infant HSCR patients (Tang et al., 2012a) and in mouse embryos (Orr-Urtreger et al., 1993). NRG1 immunoreactive signals were previously detected in myenteric ganglia of mice (Crone et al., 2003) and normoganglionic segement of infant HSCR patients (Garcia-Barcelo et al., 2009), however tissue discrimination in both mRNA and immunohistochemical studies was not carried out, so that to our knowledge this study presents the first data on site-specific NRG1 expression levels in the colon.

NRG1 protein was not only found in the neuropil and neuronal somata of enteric neurons and glial cells, but also in the neuronal and glial nucleus. Interestingly, some NRG1 isoforms contain putative nuclear targeting sequences (Holmes et al., 1992), that translocate to the nucleus and alter gene expression as shown for the type I NRG1 isoform heregulin β1, whose translocation generates proapoptotic effects in breast cancer cells (Li et al., 1996; Weinstein and Leder, 2000) and for cysteine-rich domain isoforms of NRG1 in sensory neurons of mice spinal ganglia (Bao et al., 2003). The authors demonstrated that nuclear NRG1 represses the expression of several apoptotic regulators, resulting in diminished death of neuronal cells *in vitro*.

Both ERBB2 mRNA expression and immunoreactive signals, that co-localize with neural structures, were only found to a minor extent in the human myenteric plexus and as dense punctate staining in the neuronal somata and neuropil, as shown before in dorsal root ganglion (DRG) sensory neurons (Pearson and Carroll, 2004). ERBB3 immunoreactivity was found throughout neuronal somata and the neuropil and mRNA expression was similar to that observed in muscle cells. Despite its function for neural crest differentiation and sympathetic nervous system development (Britsch et al., 1998), the presence of ERBB2 and ERBB3 immunoreactivity and mRNA expression within the postnatal ENS indicates an involvement of the NRG1-ERBB2/ERBB3 signaling system in maintaining and modifying neuronal synapses during adulthood. This could be likely, since it was recently found, that: (i) NRG1 upregulates the expression of nAChR via the ErbB2/ErbB3-PI3K-MAPK signaling cascade in adult autonomic ganglia (Kim et al., 2013); and (ii) NRG1 was observed to regulate the expression of nicotinic acetylcholine receptors and formation of synapses (Yang et al., 1998) and to induce the regulation and expression of post-synaptic GABA receptors in the CNS (Flames et al., 2004).

Also in nerve fibers of the circular musculature of the human colon, slight NRG1 immunoreactivity was found and confirmatory to that, NRG1 immunoreactivity was detected in enteric nerve fibers of single neurons and ganglion-like aggregates. In the CNS, it is known, that neuronal axons express NRG1 type III to regulate myelination (Nave and Salzer, 2006). However, at the neuromuscular junction it is thought that motoneuron-derived NRG1 type I acts on skeletal muscle fibers to increase expression and clustering of cholinergic receptors (Jo et al., 1995; Sandrock et al., 1997; Loeb et al., 2002). In the PNS, NRG1 was found at synapses on phrenic α-motoneurons (Issa et al., 2010) and concentrated in the postsynaptic subsurface cistern of c-bouton inputs to α-motoneurons (Gallart-Palau et al., 2014). In line with this, NRG1 co-localizes with the synaptic vesicle marker SNAP-25 in enteric nerve cell cultures, pointing to NRG1 expression in or near synaptic varicosities of the ENS.

### Expression and Localization of NRG1, ERBB2 and ERBB3 in the Colonic Musculature

In the circular and longitudinal muscle layers NRG1 mRNA expression were detected and NRG1 immunoreactivity in the circular musculature could also be observed in some but not all muscle cells. Similar to that, skeletal muscle cells express NRG1 transcripts (Jaworski and Burden, 2006) and it is suggested that muscle-derived NRG1 is involved in synapse-specific AChR transcription (Rimer, 2003) and myotube formation (Kim et al., 1999). However, its role in the smooth musculature remains unclear.

The main source of ERBB2 mRNA expression was found in the circular musculature, whereas ERBB3 was similarly expressed in the myenteric plexus and the musculature. Confirmatory to that, ERBBB2 displayed much more signals in the musculature as in nerve fibers, however ERBB3 showed minor signals in the musculature. Both, ERBB2 and ERBB3 expression was shown before in muscle tissue e.g in human bladder (Borer et al., 1999) and ErbB2 but no ErbB3 expression was found in vascular smooth muscle cells (Iivanainen et al., 2003). Regarding its diverse functions, it is likely, that ErbB2 has additional roles in the musculature e.g., signaling elicited by other factors, such as EGF (Carraway and Cantley, 1994), endothelin (Daub et al., 1996), and cytokines, such as IL-6 (Qiu et al., 1998).

In adult skeletal muscle and in C2 myoblasts and myotubes ErbB2 and ErbB3 expression was previously shown, and it was also reported, that NRG1 stimulates tyrosine phosphorylation of ErbB2 when co-expressed with ErbB3. Thus, the authors conclude, that ErbB3 is the predominant NRG1 receptor in muscle cells and that a complex of ErbB2 and ErbB3 mediate NRG1 signaling in skeletal muscle (Jo et al., 1995). However, NRG1-ERBB2/ERBB3 signaling in the smooth musculature remains unclear, but its presence in the adult human colon points to a putative signaling role in the smooth musculature.

### NRG1 as Neurotrophic Factor and Interaction with other Neurotrophic Factors

In postnatal enteric nerve cultures, we observed, that NRG1 significantly enhanced total neurite length, branching and number of ganglion-like aggregates suggesting a critical role for NRG1 as a trophic factor not only in the developing ENS but also during adulthood as shown before for the neurotrophic factor GDNF (Böttner et al., 2013). In the CNS and PNS it is suggested, that NRG1 signaling is also critical for the normal development and distribution, since cortical interneurons depend on NRG1 for migration within the telencephalon toward to the cortex (Flames et al., 2004) and cultured hippocampal neurons increase neurite outgrowth, area, length, and branching when exposed to NRG1 (Gerecke et al., 2004). In dorsal root ganglia explants, NRG1 also regulates outgrowth of neurites and migration of neurons (Liu et al., 2011).

For development and maintenance of the nervous system, it is established, that two-way communication is needed between neuronal cells and target cells (Korsching, 1993; Esper and Loeb, 2009). Reciprocal communications between neuronal NRG1 and target-derived neurotrophic factors such as GDNF has been uncovered in the CNS, where GDNF stimulate the expression of NRG1 in motor neurons both *in vitro* and *in vivo* (Loeb and Fischbach, 1997; Loeb et al., 2002) and locally applied GDNF induced the rapid release of NRG1 from neurons and their axons (Esper and Loeb, 2009). We could not find any effects on the mRNA expression of NRG1 on postnatal enteric neurons, however, we found a decrease of ErbB2 and ErbB3 mRNA expression in response to GDNF treatment. Down-regulation of ErbB3 mRNA expression in response to GDNF stimulation was shown before in mouse enteric neural crest cells (Gui et al., 2013) indicating a functional interaction between NRG1 and GDNF signaling in the ENS. However, not only GDNF stimulation, but also NRG1 exposition to enteric nerve cultures resulted in decreased expression of ErbB3, an effect shown before in MCF7 cells, where ErbB3 down-regulation due to NRG1 stimulation resulted in elevated steady-state levels of ErbB3 protein (Cao et al., 2007). Thus, it seems likely that a GDNF-stimulated release of NRG1 could be finally responsible for the observed down-regulation of ErbB3 in GDNF treated enteric nerve cell cultures.

### Relevance of the NRG1-ErbB Signaling System for Enteric Neuropathies

Given the broad-range functions of the NRG1/ErbB signaling system in the nervous system during development and in the adult, NRG1 and ErbBs have been implicated in several neuronal diseases of the CNS, e.g., schizophrenia (O’Donovan et al., 2008), bipolar disorder (Goes et al., 2009) or Alzheimer’s disease (Chaudhury et al., 2003), where pathophysiological genetics such as NRG1- polymorphism (Go et al., 2005) or abnormal protein or mRNA levels of NRG/ErbB signaling components have been associated (Mei and Nave, 2014).

Interestingly, there is strong genetic and functional evidence for a role of type I NRG1s in the pathogenesis of HSCR (Tang et al., 2012a), a congenital disorder of the ENS, that is associated with a complete loss of enteric neurons (aganglionosis), since mutations in the NRG1 gene are associated with this disease (Garcia-Barcelo et al., 2009). NRG1 seems to contribute to both common and rare HSCR variants (Luzón-Toro et al., 2012) and aberrant high expression of type I NRG1 was observed in aganglionic and normoganglionic segments of patients with HSCR (Tang et al., 2012b), providing evidence for a pathological role of dysregulated NRG1 in enteric neuropathies. Thus it is assumed that distinct alterations of the NRG1-ErbB pathway do not only lead to HSCR, that display complete aganglionosis, but may also be associated with or contributed to other enteric neuropathies probably characterized by a partial loss of enteric neurons (hypoganglionosis), e.g., slow-transit constipation or diverticular disease (De Giorgio and Camilleri, 2004; Wedel et al., 2010), all disorders belonging to the group of gastrointestinal neuromuscular diseases (GINMD; Knowles et al., 2010).

Because an interaction of the NRG1 and GDNF signaling system can be supposed in the ENS, the idea of an altered NRG1 system in some GINMDs is supported by the observation of an altered GDNF system, as demonstrated so far not only for HSCR (Butler Tjaden and Trainor, 2013) but also for diverticular disease, where a lack of the neurotrophic factor GDNF and its receptors RET and GFRα1 was recently observed (Böttner et al., 2013).

In conclusion, we have demonstrated the presence of the NRG1-ErbB2/ErbB3 system in main colonic neuromuscular components and illustrated that NRG1 promotes growth and differentiation of postnatal enteric neurons arguing for a role of NRG1 in the maintenance of the ENS during postnatal life. Further, down-regulation of ErbB receptor expression in response to GDNF stimulation points to an interaction of both signaling systems in enteric neurons. Thus, these findings could serve as a basis to further investigate whether altered NRG1-ErbB signaling may be linked to intestinal diseases associated with enteric neuropathies. Possible new findings could also contribute to more sophisticated diagnostics or therapies.

## Author Contributions

MB was responsible for study design, acquisition and interpretation of data, data analysis and writing of the paper, CL and FC were responsible for acquisition and interpretation of data and critically revising of the manuscript, TB and J-HE contributed to the human material acquisition, TW and MB critically revised the manuscript and wrote the grants financing the study.

## Funding

This work was supported by research grants from the Deutsche Forschungsgemeinschaft (DFG, WE 2366/4-2). The funding source has no influence in the design of the study, management of the data or writing of the paper.

## Conflict of Interest Statement

The authors declare that the research was conducted in the absence of any commercial or financial relationships that could be construed as a potential conflict of interest.
